# ROSBASE1.0: a comprehensive database of reactive oxygen species (ROS): categorization of cell organelles, proteins, taxonomy, and diseases based on ROS-related activities

**DOI:** 10.1093/database/baaf069

**Published:** 2025-10-31

**Authors:** Sharayu Ghodeswar, Debashree Bandyopadhyay

**Affiliations:** Department of Biological Sciences, Birla Institute of Technology and Science, Pilani, Hyderabad Campus, Jawahar Nagar, Kapra Mandal, Medchal District, Hyderabad, Telangana, 500078, India; Department of Biological Sciences, Birla Institute of Technology and Science, Pilani, Hyderabad Campus, Jawahar Nagar, Kapra Mandal, Medchal District, Hyderabad, Telangana, 500078, India

## Abstract

The reactive oxygen species (ROS) produced by various cell organelles often relate to pathophysiology or pathogenesis. Information on ROS-producing, scavenging, and regulatory proteins from various taxonomic kingdoms and organelles, enzyme classes, and diseases was scattered across the literature. Consolidation of all information would facilitate the researchers to understand ROS-related pathology and possible therapeutics. For the first time, we developed a secondary database, ROSBASE1.0, consolidating ROS protein features, taxonomy, diseases, and interactome data. Data sources were PubMed, UniProt, STRING, and Google Scholar databases. Notably, 81.5% of the reported data were experimentally curated. The ROS mechanism was elucidated for 79.2% of entries based on text-mining from the literature. A total of 2494 experimentally annotated entries (UniProt IDs) were curated in the database. Multiple PDB IDs correspond to individual UniProt IDs, resulting higher number of PDB entries (*n* = 9174). The database reported 2447 genes, 16 cell organelles, 395 organisms, and 218 disease entries (corresponding to 143 proteins) classified into 14 categories. In this database, proteins were classified based on taxonomic kingdoms, enzyme classes, and diseases. The keywords to search the database were—UniProt ID, PDB ID, cell organelle, organism name, and gene name. The tabular results depict all the above features, in addition to the interactome network. The database (https://rosbase.bits-hyderabad.ac.in/) for the first time quantitatively demonstrated the preferences of lower organisms towards ROS-scavenging versus ROS production by higher organisms. Categorization of different diseases according to their ROS involvement was also reported here for the first time. This database could potentially serve as a user guide to ROS Biology.

## Introduction

The highly reactive oxidizing agents, containing at least one atom of oxygen and unpaired electrons, are referred to as reactive oxygen species (ROS), e.g. singlet hydroxyl, singlet oxygen, hydroperoxyl radicals, [1, 2], hydroxyl radicals (OH), hydrogen peroxide (H_2_O_2_), superoxide radical anion (O_2_^−^), ozone (O_3_) [3], etc.; these are the byproducts of incomplete molecular oxygen decomposition. ROS is an important component for cellular processes at low to moderate concentrations, but could be lethal due to overproduction. Thus, ROS can be deleterious to cells but can also be an integral part of cell survival and adaptive signalling [4]. Considering the dual effects of ROS, previously termed ‘oxidative stress’ has been redefined as ‘oxidative eustress’ and ‘oxidative distress’ [5, 6]. ‘Oxidative eustress’ was defined as adaptive cell responses to ROS that induce allostasis, followed by the reversible and site-specific oxidation or reduction of protein-cysteine. ‘Oxidative distress’ was defined as overproduction of ROS beyond the beneficial physiological range, thus leading to cell damage and disease. A few examples of ‘oxidative distress’ are, ROS induced cell damage by altered signalling pathways [7, 8] cancerous growth by DNA damage and altered cell signalling [9], ROS-induced pathological stages, such as cardiovascular diseases [10], myocardial infarction/ischaemic myocardium [11], metabolic syndrome, insulin resistance, obesity, diabetes [12], neurodegenerative diseases [13], etc. The most common source of ROS is the respiratory complexes I and III in mitochondria, through which most of the electrons are transported to the terminal electron acceptor, molecular oxygen. Recently, unconventional sources of ROS in mitochondria were identified, which were isopotential groups of two classes, NADH/NAD and CoQH2/CoQ ( Fig. 1 of [4]). These groups can produce more ROS than Complex I. Around 0.2%–2% of the electrons in electron transport chain (ETC) leak out and form ROS molecules, such as superoxide and hydrogen peroxide [14]. The mitochondrial permeability transition pores contribute to maintaining mitochondrial ROS homeostasis [15]. Mitochondrial uncoupling proteins (UCPs) play an important role in controlling ROS production by modulating the proton gradient across the inner mitochondrial membrane. UCP2 and UCP3 decrease mitochondrial membrane potential through mild uncoupling, effectively reducing the electron leak that drives superoxide generation and its consequences [16–18]. UCPs could also regulate oxidative stress through calcium transport and lipid hydroperoxide export [19–21]. In addition to respiratory complexes, a few dehydrogenases in mitochondria generate ROS, such as Alpha-ketoglutarate dehydrogenase (α-KGDH), pyruvate dehydrogenase (PDH), and branched-chain α-keto acid dehydrogenase (BCKDH). All these complexes share similar structures and belong to the Alpha ketoacid dehydrogenase complex family, catalyse the oxidative decarboxylation of specific α-ketoacids [22]. α-KGDH and PDH are involved in both ROS production and scavenging. The α-KGDH produces ROS, especially under NADH/NAD⁺ imbalance, promoting mitochondrial dysfunction and Parkinson’s disease [23]. Under low NADH/NAD⁺ conditions, both KGDH and PDH produce ROS via reverse electron transfer (RET), thus amplifying RET from complex II to I [24, 25]. Under metabolic stress, PDH is inhibited by PDH kinase [26], thus reducing mitochondrial respiration and ROS production. ROS can be produced in many more cellular compartments, other than mitochondria, such as cytoplasm, membranes, endoplasmic reticulum, lysosomes, peroxisomes, etc. [27]. The most common ROS species detected in the cell are hydrogen peroxide (H_2_O_2_), superoxide (^•^O₂^–^), hydroxyl radicals (^•^OH), and singlet oxygen (^1^O_2_), produced in different cellular compartments (Table 1). For example, superoxide radical (^•^O₂^–^) is produced within mitochondria by Complex I and Complex III and scavenged to molecular oxygen (O₂) by superoxide dismutase (SOD) [28]. Hydroxyl radicals (^•^OH) are produced in mitochondria and cytosol due to the loss of electrons from water molecules [29]. Hydrogen peroxide (H₂O₂) is formed in mitochondria, cytosol, and nucleus and converted to water and oxygen, mainly, by the catalase enzyme [30]. Singlet oxygen (¹O₂) is produced in the chlorophyll due to the photosensitization of ground-state oxygen (³O₂) [31]. The hydroxyl radical (•OH) reacts with carbon and molecular oxygen to produce the peroxyl radical ROO^•^ that undergoes a chain reaction to produce hydroperoxide ROOH and alkoxyl radical RO^•^ [32]. The term ‘ROS’ also, generously, includes nitrogen-containing reactive species, termed as reactive nitrogen species; e.g. nitric oxide (NO^•^), anion (NO^−^), etc. The ROS molecules are linked to various redox and cell signalling pathways, thus contributing to various cellular activities and cellular homeostasis [14, 15, 33]. In fact, ROS (particularly, H_2_O_2_) plays a central role in the ‘Redox Code’ that regulates biological operations; the term was introduced analogous to ‘genetic code’, ‘epigenetic code’, ‘histone code’, etc. [34]. ‘Redox code’ consists of four principles, briefly, those are, (i) use of reversible electron carriers, namely NAD+/NADH and NADP+/NDAPH to provide organization of metabolism, that constitutes activation and deactivation of redox cycles (redox sensing), (ii) metabolism is linked to protein tertiary structures, interaction network, activities, etc., through kinetically controlled redox switches [various cysteine post-translational modifications (Cys-PTMs)] in the proteome, (iii) redox sensing, especially involving H_2_O_2_, support spatiotemporal sequencing in differentiation and life cycles of cells and organisms, and (iv) redox networks form an adaptive system to respond to the environment from sub-cellular organelles to the levels of cell and tissue organization. Hydrogen peroxide (pivotal in ‘Redox Code’) levels within the cell are predominantly regulated by Peroxiredoxins (Prxs), a ubiquitous family of cysteine-dependent peroxidase enzymes [35–39]. At higher exposure to H₂O₂, the cysteine residues of Prxs undergo oxidation to sulfinic acid, causing temporary inactivation of the enzymes [40–42].

**Figure 1.**
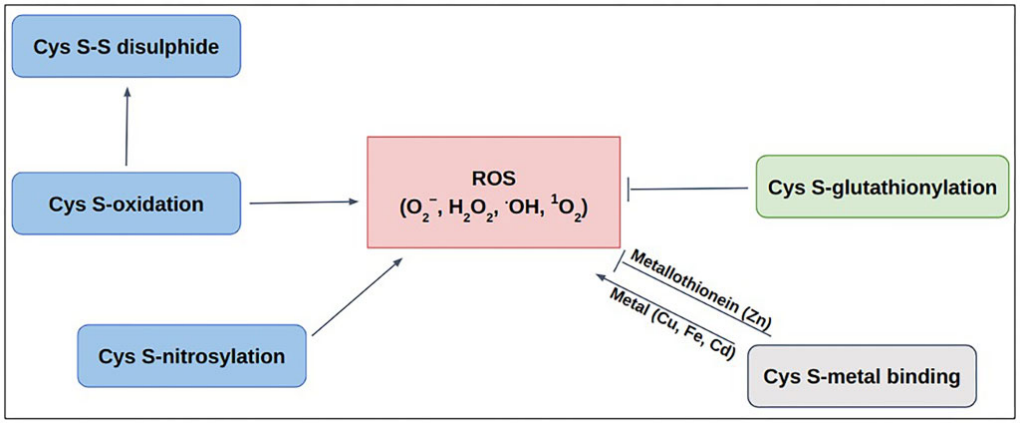
Schematic diagram of Cys-PTMs involved in ROS-related activities: ROS scavenging (—|) and ROS producing (→).

**Table 1.** Production of ROS in different cellular compartments.

ROS species	Source	Reactions
Superoxide radical (^•^O^−^_2_) [28]	Mitochondria associated with Complex I and Complex III	O_2_ →e^−^ + ^•^O^−2^ M^(^*^n^*^+1)+^−SOD + O^−2^ → M*n*^+^−SOD + O_2_ (superoxide dismutase) (Mn- Cu (*n *= 1); Mn (*n *= 2); Fe (*n *= 2); Ni (*n *= 2))
Hydroxyl radical (^•^OH) [29]	Mitochondria, cytosol	H_2_O →e^−^ + **^•^**OH (UV/x-rays) M*n*^+^−SOD + O^−2 ^+ 2H^+^ → M^(^*^n^*^+1)+^− SOD + H_2_O2
Hydrogen peroxide (H_2_O_2_) [30]	Mitochondria, cytosol, nucleus	2H_2_O_2_ → 2 H_2_O + O_2_ *(catalase)* 2GSH + H_2_O_2_→ GS–SG + 2H_2_O *(Glutathione peroxidase)*
Singlet oxygen (^1^O_2_) [31]	Chlorophyll	^3^O_2_ →^1^O_2_ (Photosensitizer)

### ROS-related protein categories

ROS-related protein activities are diverse, ranging from ROS production, scavenging, and regulatory activities. Here, we categorized ROS-related proteins into four groups, namely, ROS-producing, ROS-scavenging, ROS-producing-and-scavenging, and ROS-indirect involvement.

#### ROS-producing

ROS production is mediated by enzymes such as NADPH oxidase (NOX) and other mitochondrial enzymes. Specific examples are, (i) macrophage proteins interact with the NOX subunit NCF1 and activate NOX-dependent ROS generation as a defence mechanism, e.g. against sepsis [43], (ii) Vasculostatin-40 enhances the binding of Gram-negative bacteria and prompts macrophages to generate ROS (microbicidal functions) [44], (iii) the ERO1-like protein facilitates ROS formation during protein disulfide production by transferring electrons to molecular oxygen [45], (iv) Thrombospondin-1 enhances ROS production in endothelial cells and contributes to cellular aging [46–48], and (v) in the hypothalamus region, Leptin protein pro-opiomelanocortin (POMC) neurons increase neutrophil chemotaxis and oxygen radical release [49].

#### ROS-scavenging

ROS scavenging is mediated by antioxidant enzymes, such as SOD, catalase (CAT), peroxidase (GPX), etc. Specific examples are, (i) catalase activates peroxisomal oxidases to convert hydrogen peroxide into water and oxygen [50], (ii) SOD neutralizes toxic radicals [51, 52], (iii) aquaporin-8, regulates ROS signalling by absorbing hydrogen peroxide [53].

#### ROS-producing-and-scavenging

Some proteins exhibit dual roles of ROS production and scavenging and maintain cellular homeostasis, few examples are cited. (i) Glycerol-3-phosphate dehydrogenase that balances ROS by shuttling mitochondrial glycerol-3-phosphate and maintains cellular NADH/NAD + ratio [54], (ii) cellular communication network factor 1 (CCN), produces ROS and also contributes towards the positive feedback loop by oxidative stress [55], (iii) Parkinson’s disease-associated protein, PARK7 homolog, maintains mitochondrial ROS and glucose homeostasis in pancreatic islets in response to age and nutrition, and protects pancreatic beta cells from inflammation or cytotoxicity-induced cell death [56].

#### ROS-indirect-involvement

ROS is crucial for maintaining cellular homeostasis through a cascade of reactions, chaperone activities, etc. A few examples, (i) thioredoxin reductase-2 regulates mitochondrial redox homeostasis and ROS levels [57], (ii) mitochondrial protein adenylyl transferase, SelO, maintains redox haemostasis by contributing towards Adenylation (involving adenosine 5’-monophosphate) and protein S-glutathionylation; these processes modulate several cellular oxidative stress responses [58], (iii) PARK7 is also indirectly involved in ROS activity by chaperoning copper ions to the active site of SOD protein [59], (iv) peroxisomal heat shock protein acts as chaperone at elevated temperatures and generates ROS [60], and (v) early secretory antigenic target triggers a cascade of events—activation of ERK—p38 mitogen-activated protein kinase signalling pathways—ROS production [61].

### Involvement of cysteine amino acid in ROS activity

Redox modifications, mainly the reversible oxidation of cysteine residues in proteins, are critical for preventing oxidative distress. Those act as redox switches, allowing proteins to sense and adapt to oxidative signals, *a priori*, permanent damage, thereby maintaining cellular balance [62–64]. Most of the ROS-associated proteins have cysteine (Cys) at their active sites. Different post-translational modifications of Cys, namely, metal binding [65, 66], disulfide [67], S-nitrosylation [68], glutathionylation [68], and other oxidative post-translational modifications (OxPTMs) [69], are involved with ROS in various ways (Fig. 1). Cysteine metal-binding was observed in iron–sulphur clusters, haem, and zinc finger motifs, which maintain cellular redox balance and protect against oxidative stress [65]. S-oxidation, S-glutathionylation, and S-nitrosylation of Cys, produce ROS in mitochondria; whereas S-glutathionylation prevents oxidative stress by mitigating ROS production in key sources, such as α-KGDH and PDH, etc. These dehydrogenases are inhibited by ROS as a consequence of OxPTMs such as S-glutathionylation and S-nitrosylation that result in reduced NADH and ATP production, accumulation of TCA intermediates [70]. S-nitrosylation of the lipoic acid moiety on KGDH inhibits its enzymatic activity, thereby reducing NADH production and downstream electron flux, ultimately decreasing ROS generation from the respiratory chain [71]. Similarly, S-glutathionylation of KGDH also results in its reversible inhibition, thus protecting against irreversible oxidative damage during oxidative stress [72]. S-nitrosylated proteins in mitochondria (SNO-proteins) inhibit respiratory Complex I, ROS production, and a series of mitochondrial processes [73]. OxPTM includes ROS-mediated oxidation of cysteine thiol (−SH) to sulfenic acid (−SOH). Sulfenic acid can be further oxidized to sulfinic acid (^−^SO₂H) and sulfonic acid (^−^SO₃H). These modifications are often reversible and involved in signalling that can alter protein function or act as a precursor to further oxidative modifications [74–76]. However, only selective cysteine residues undergo ROS-mediated modifications depending upon their physiological requirements and local redox environment.

## Methodology

The information on ROSBase1.0 was gathered from different databases, namely, UniProt (www.uniprot.org), PDB (www.rcsb.org), PubMed (https://pubmed.ncbi.nlm.nih.gov/), and STRING (https://string-db.org/).

### ROS-related data curation from different databases

#### UniProt database

The ROS-related data were primarily curated from the UniProt database dated 17.04.2025. The search was exhaustive for the given keywords ‘reactive oxygen species’.

The initial search resulted in 86 133 entries. The search results were further filtered using ‘Reviewed’ (experimental and computer-predicted) keyword, which returned 2494 protein entries. Two viral proteins were excluded due to the ambiguity of the ROS source, whether it is from the host or the virus. Hence, a total of 2492 UniProt IDs were considered. The customized search results were downloaded based on the Application Programming Interface (API) of UniProt, with the following features, protein name, gene name, UniProt ID, organism, function, sub-cellular location (cell organelle), PubMed ID, and PDB ID.

#### PubMed database

A total of 12 469 PubMed IDs were retrieved from the abovementioned UniProt API, and 9766 articles were downloaded from the PubMed database. The articles not available were downloaded using PMC IDs from the PMC database (*n* = 861). A few more articles were downloaded from respective journal homepages (*n* = 148). A total of 921 articles could not be downloaded due to restricted permissions. Hence, a total of 10 775 articles were obtained out of 12 469 PubMed IDs.

#### Text mining of the research articles

The downloaded articles were subject to text mining using an in-house Python script. The Python modules implemented in this code were: *regular expression (regex/re)* (pattern matching); *glob* (finding files in the directory); *csv* (handling comma-separated-value files); *fitz* (extracting text from PDF); and *tqdm* (indicating progress bar). A list of PDF filenames was extracted using the glob module. Opening and reading each PDF file, progress monitoring within a for loop was performed using *tqdm* module. The page numbers were extracted using *fitz* module. A nested ‘for loop’ was iterated over the number of pages (per PDF file) to extract text from each page. The search keywords within the page were ‘Reactive’, ‘oxygen’, and ‘species’, using regular expressions (*regex* module). Approximately, ±500 characters were collected around the matched keywords and the contextual snippet was saved in a CSV file, including the filename and page number. The saved CSV file stored ROS-related protein information, diseases, protein functions, and mechanisms. All the data gathered from 6082 articles were compiled into a ‘JSON’ file.

#### STRING database

The STRING database was searched for interactome data, using UniProt IDs (*n* = 2494), resulting in 1951 entries.

**Figure 2.**
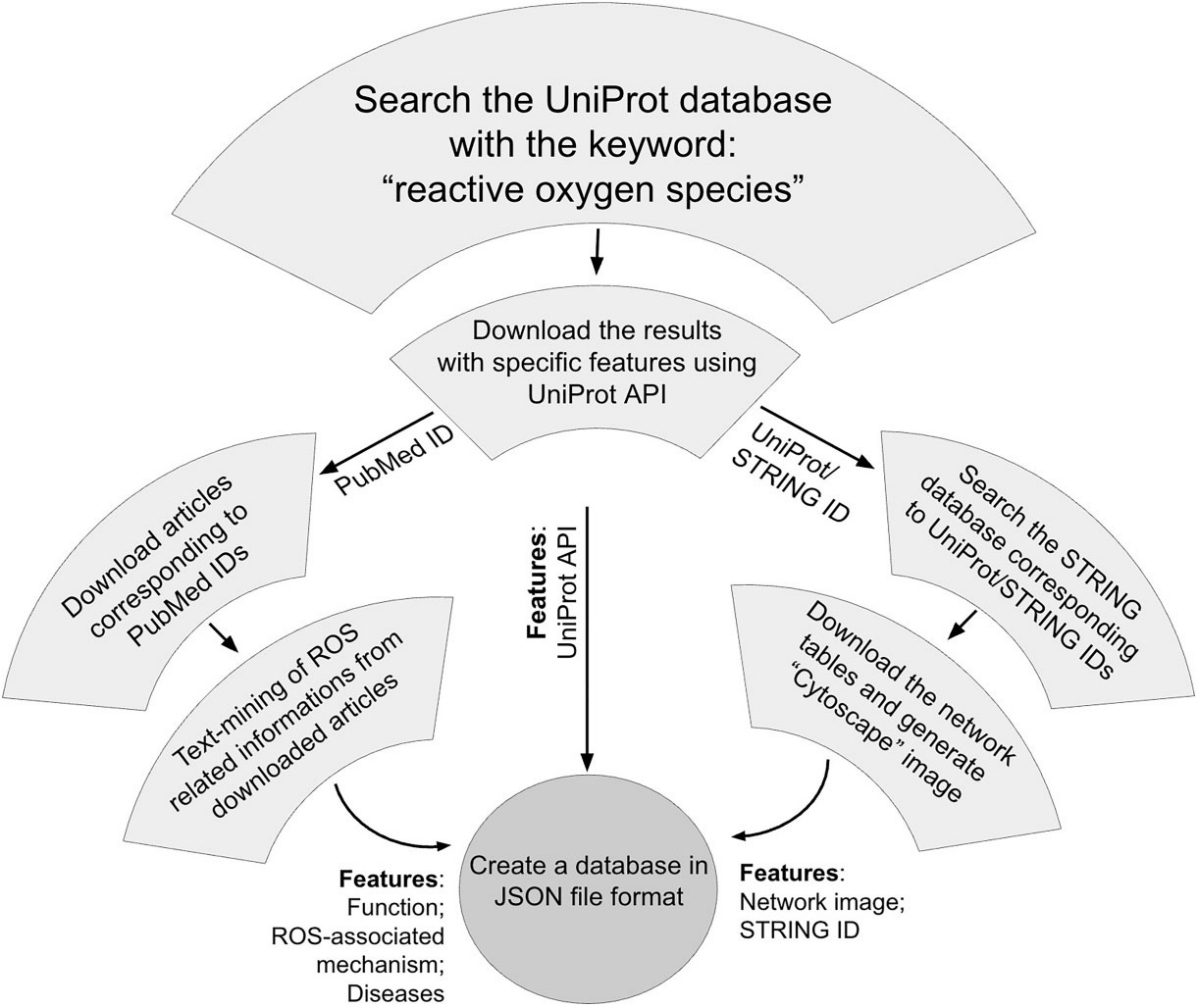
Flowchart of ROSBase curation.

Each of these string entries corresponds to a single ‘central protein’ in the current study, in contrast to the STRING database, where interactions of multiple proteins with multiple partners are described. We have filtered a single protein (entry) and generated the interactome network around it. All the partners, corresponding to that single protein, were exported as a tab-separated-value (TSV) file. Each TSV file was imported into the Cytoscape software (v 3.10.2) for display. The central protein and the interacting partners were colored differently, and the image was saved as a picture (.png) format. All these images were saved in a static folder. Image names and interactome IDs were added in two separate columns in the ‘JSON’ file that completes the ‘ROSbase’ database. The flowchart of the database is shown (Fig. 2).

#### Web server development

The ROSBase1.0 database web server was developed using the Flask lightweight web framework on the Ubuntu 20.04 platform, implemented in Python. This web server offers a user-friendly graphical user interface (GUI) to search the database. The following software packages, environments, and libraries were installed for this web application, namely, Python3 (v 3.8.10), Flask (v 2.3.2), Gunicorn (v 21.2.0), and Nginx (v 1.18.0). The deployment process included Flask application setup, Green Unicorn (Gunicorn) and Nginx configuration, and ‘systemd’ service unit creation. Nginx, the high-performance web server and reverse proxy, forwards the requests from clients to the local Flask application, recruited as a production server by Nginx. The Flask production server ensures stability, scalability, and optimized performance. Gunicorn Web Server Gateway Interface communicates between the HTTP server front end and back end. The application was encrypted with SSL certificates (https://certbot.eff.org/), firewall settings were adjusted; accessibility and functionality were verified.

#### Backend of the webserver

**Figure 3.**
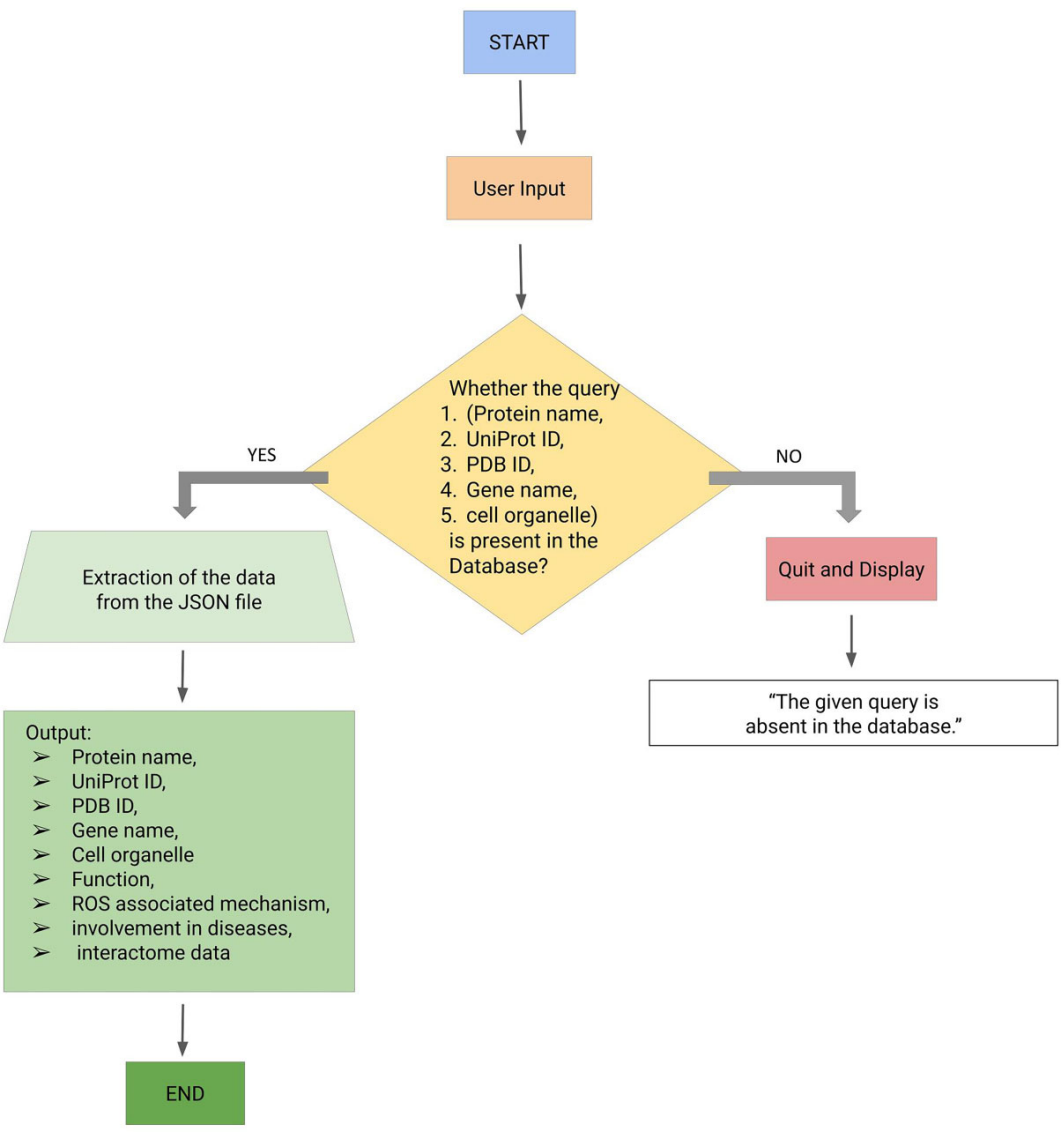
Workflow (integrating backend and frontend) of ROSBase1.0 webserver.

The backend of the ROSBase server was based on an in-house Python code, input to that is a user-defined query (any one of the following—protein names, UniProt IDs, PDB IDs, cell organelles, or gene names) and output is ROS-related information from the JSON file. There was a check whether the query was present in the database; if ‘yes’, the results would be returned from the JSON file, if ‘no’, an error message would be displayed with termination (Fig. 3). The result returns the following features in tabular form, Protein name, UniProt ID, PDB ID, gene name, function, ROS-associated mechanism, diseases, and interactome data (provided the string ID was available for that particular entry). If String ID was not available it would be redirected to the STRING homepage. The interactome image would only be displayed for a single output result and not for multiple outputs. For example, the query name, mitochondria, yielded multiple outputs and the Cytoscape images or STRING ID would not be displayed.

## Results and discussion

### Data statistics of ROSBase

The database consisted of 2494 entries (UniProt IDs), 2182 genes, 6959 PDB IDs, and 10 775 PubMed entries. The gene names were fewer than the total number of entries because some genes were not reported corresponding to the proteins. The number of PDB IDs exceeded the number of entries due to multiple crystal structures for certain proteins. A total of 17 cell organelles, 262 organisms, and 243 diseases (14 categories) were reported in the database. All the taxonomic groups were well represented in the current study—Animalia (*n* = 1569), Plantae (*n* = 359), fungi (*n* = 194), Protista (*n* = 38), and bacteria (*n* = 334). The diseases were only represented for Homo sapiens (*n* = 143).

### Validation of the data

(i) UniProt entries:
Out of 2492 UniProt entries (protein sequences), 1469 sequences were identified at the protein level, using complete or partial Edman sequencing and identification by mass spectrometry, X-ray, and Nuclear Magnetic Resonance (NMR) structure, and detection of the proteins by antibodies (www.uniprot.org). A total of 563 sequences were derived from the transcripts, experimentally obtained from mRNA expression. The remaining 462 entries were computationally predicted. Thus, 81.5% of the entries in the database were authenticated by experiments.
(ii) From literature:
A total of 10 775 articles were downloaded from PubMed and other sources. Using text-mining, only 6082 articles have produced information based on the keywords, ‘Reactive’, ‘oxygen’, and ‘species’. These 6082 articles correspond to 1974 UniProt entries. Thus, for 79.2% of entries, the ROS mechanism was elucidated.

### ROS-related protein categories

ROS-related proteins were broadly categorized into four different groups: (i) ROS-scavenging (998 entries), (ii) ROS-producing (525 entries), (iii) both ROS-producing and scavenging (201 entries), and (iv) indirectly involved with ROS-proteins (770 entries). The largest group consisted of the ROS scavenging category, presumably because cells protect themselves against oxidative damage.

ROS-associated proteins were further classified based on cell organelles, enzyme class, taxonomy, and diseases.

### Cell organelles

All the 2494 entries were classified into 16 cell organelles that were involved with ROS [77]. Many of these cell organelles were engaged in cross-talk via physical or functional contacts (known as membrane contact sites (MCSs), crucial for the redox activity of the cell [78]. The physical contacts between the outer membrane of the mitochondria and the endoplasmic reticulum, known as mitochondria-associated membranes (MAMs) [79], transfer metabolites, such as lipids, proteins, etc. [80]. These MCS and MAMs are enriched with redox-regulatory proteins. Similarly, peroxisomes and mitochondria also crosstalk by exchanging molecules. For example, the NADH produced in peroxisomes, as a part of beta-oxidation, is transported back to mitochondria for oxidation [80]. All these ROS-related processes were physiologically essential to regulate homeostasis.

**Figure 4.**
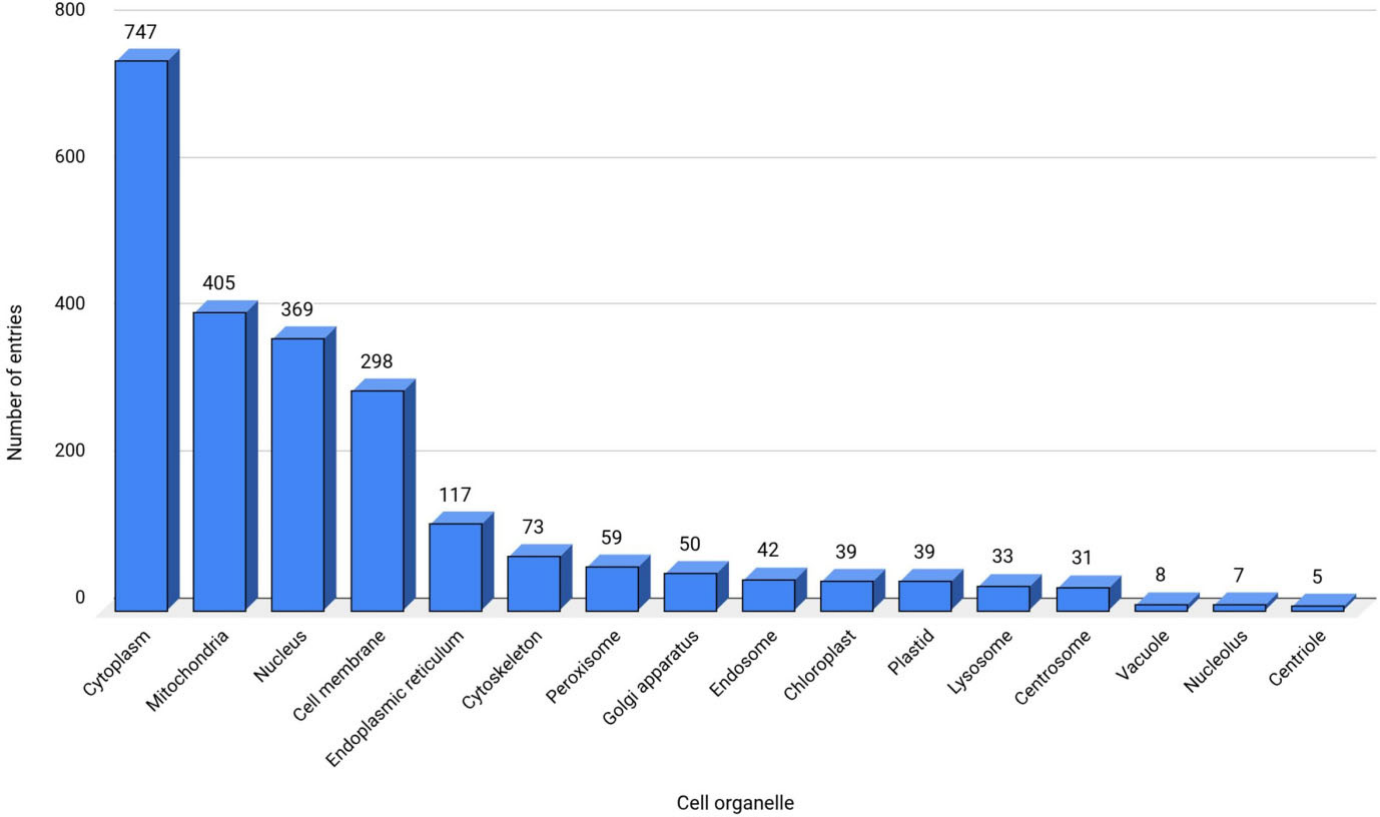
Data statistics for cell organelles. ‘Not reported’ sub-cellular location, termed here as a separate category is not shown.

Cytoplasm contains the maximum entries (747 entries) and is identified as one of the main contributors of ROS in this database (Fig. 4). The main sources of ROS in the cytoplasm are dual oxidases (DUOXI-II) and transmembrane NOXs, which produce O_2_^–^ by reducing molecular oxygen [81]. Mitochondria are the second-largest (405 entries) source of ROS in the database, where electrons escape from the final electron acceptor molecule, oxygen, and leak out from the ETC [82]. The third largest contributor of ROS in the database are nucleus, DELLA protein RGL2 (GRAS family protein 15) that promotes stress tolerance by acting on the ROS scavenging mechanism and prevents stress-induced ROS accumulation [83].

### Kingdom classification

**Figure 5.**
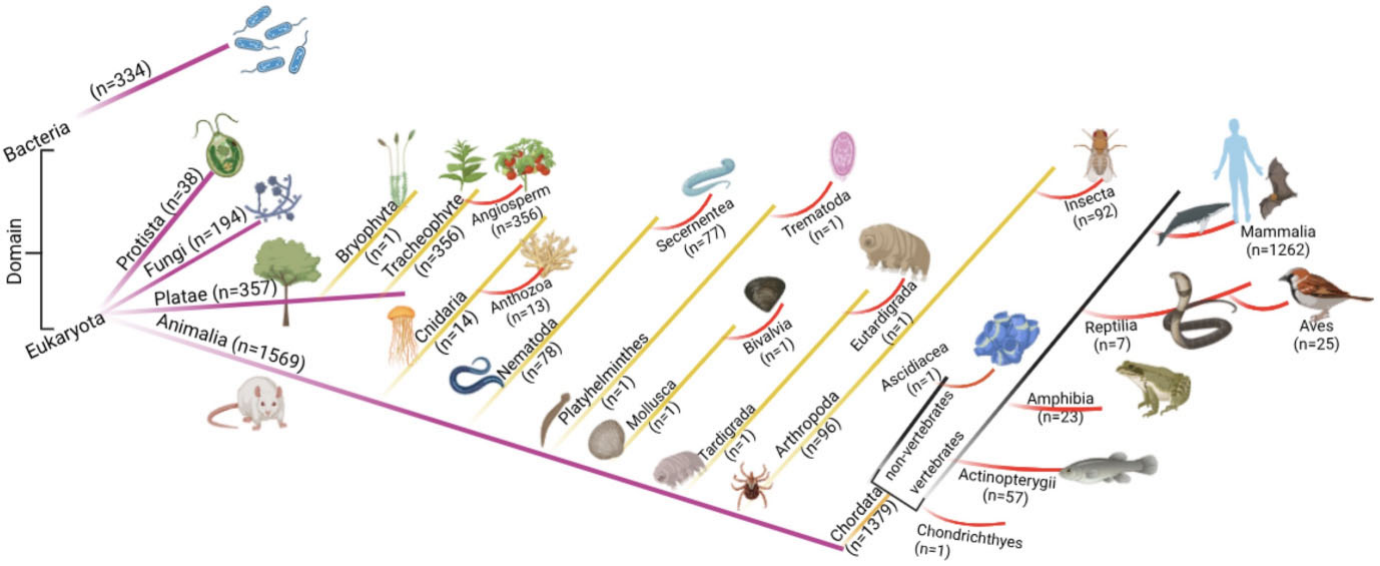
The taxonomic classification of the database. Numbers of entries in each category are shown.

The database was further classified based on the domain classification [84, 85]. The current study has non-zero entries for only two domains, Eukaryota (*n* = 2160) and Bacteria (*n* = 334). Domain Eukaryota consisted of Protista (*n* = 38), fungi (*n* = 194), Plantae (n = 359), and Animalia (*n* = 1569). The details of the taxonomy per kingdom were represented schematically (Fig. 5).

Kingdom Animalia has seven phyla and thirteen classes. Kingdom Plantae has two phyla, Bryophyta (*n* = 1) and Tracheophyte (*n* = 356); all Tracheophyte belong to angiosperm class.

**Figure 6.**
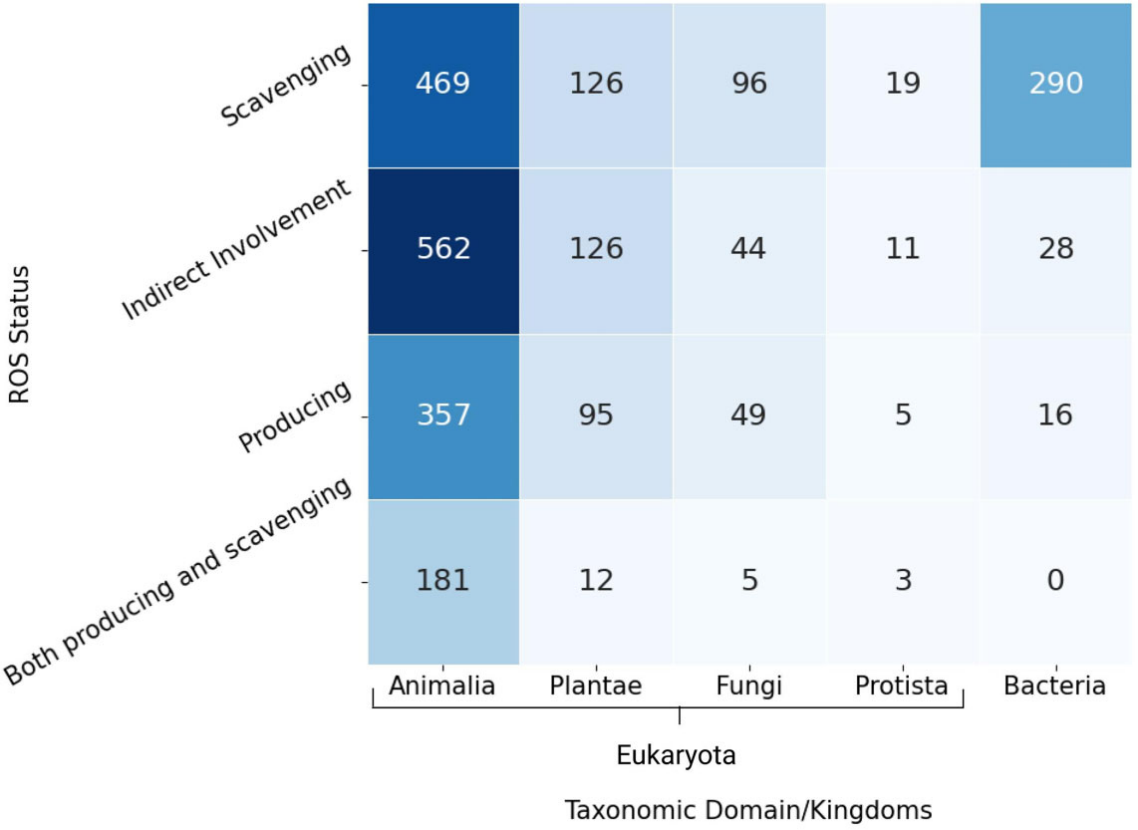
Heatmap of four different ROS-related categories in taxonomic groups based on domain [84, 85].

All the entries under the taxonomic classification exhibited ROS-related activities, although at different degrees. The Animalia kingdom hosted almost equal percentages of the ROS-producing, and ROS-scavenging proteins, and a least from ROS-indirect involvement (Fig. 6). In contrast, Kingdom Plantae has a significantly lower percentage of ROS-producing proteins (almost half) compared to ROS-scavenging proteins; although ROS-scavenging and ROS-indirect involvement entries were equivalent in number. These observations indicated the major involvement of ROS-scavenging and ROS-mediated entries in plants. A few examples of ROS-mediated physiology in plants—(i) In *Arabidopsis thaliana* (Mouse-ear cress), secreted transmembrane peptide 4 (Phytocytokine STMP4) regulates physiological and antimicrobial activities by inhibiting growth and modulating the response to ROS [86], (ii) 3-hydroxyacyl- [acyl-carrier-protein] dehydratase is involved in photorespiration, plant morphogenesis, and balances ROS levels in *Solanum lycopersicum* (Tomato) [87].

#### ROS producing

As per this database, Animalia produce the maximum amount of ROS, in terms of the number of ROS-producing entries (*n* = 357), presumably due to the complexity of the metabolic pathways and immune responses (e.g. oxidative bursts in macrophages, etc.). ROS production in Plantae (*n* = 95) is mainly related to stress signalling [88]. In bacteria (*n* = 16), ROS is generated as a byproduct of metabolic processes, such as stress response [89]. Fungi (*n* = 49) and Protista (*n* = 5) produce a minimum amount of ROS, as observed in this database.

***ROS scavenging*** is common across all organisms to mitigate ROS toxicity; highest in Animalia (*n* = 469), indicating the presence of robust antioxidant systems (e.g. catalase, glutathione peroxidase, etc.), followed by Plantae (*n* = 126), ROS scavenged via both enzymatic and non-enzymatic mechanisms [90]. ROS-scavenging is moderate to low in other kingdoms, bacteria (*n* = 290) and fungi (*n* = 96), and Protista (*n* = 19), mostly due to their stress adaptation mechanisms.

***Both producing and scavenging*** of ROS are prominent in Animalia (*n* = 181), suggesting the presence of multifunctional ROS regulators, in comparison to the other kingdoms, Plantae (*n* = 12), fungi (*n* = 5), and Protista (*n* = 3).

***Indirect involvement*** of ROS modulates different types of functional activities, such as redox signalling, transcription factors, chaperoning, etc. This indirect involvement of ROS is mostly observed in Animalia (*n* = 562), followed by bacteria (*n* = 28) and Plantae (*n* = 126), presumably due to complex signalling networks, compared to fungi (*n* = 44) and Protista (*n* = 11).

### Enzyme class

**Figure 7.**
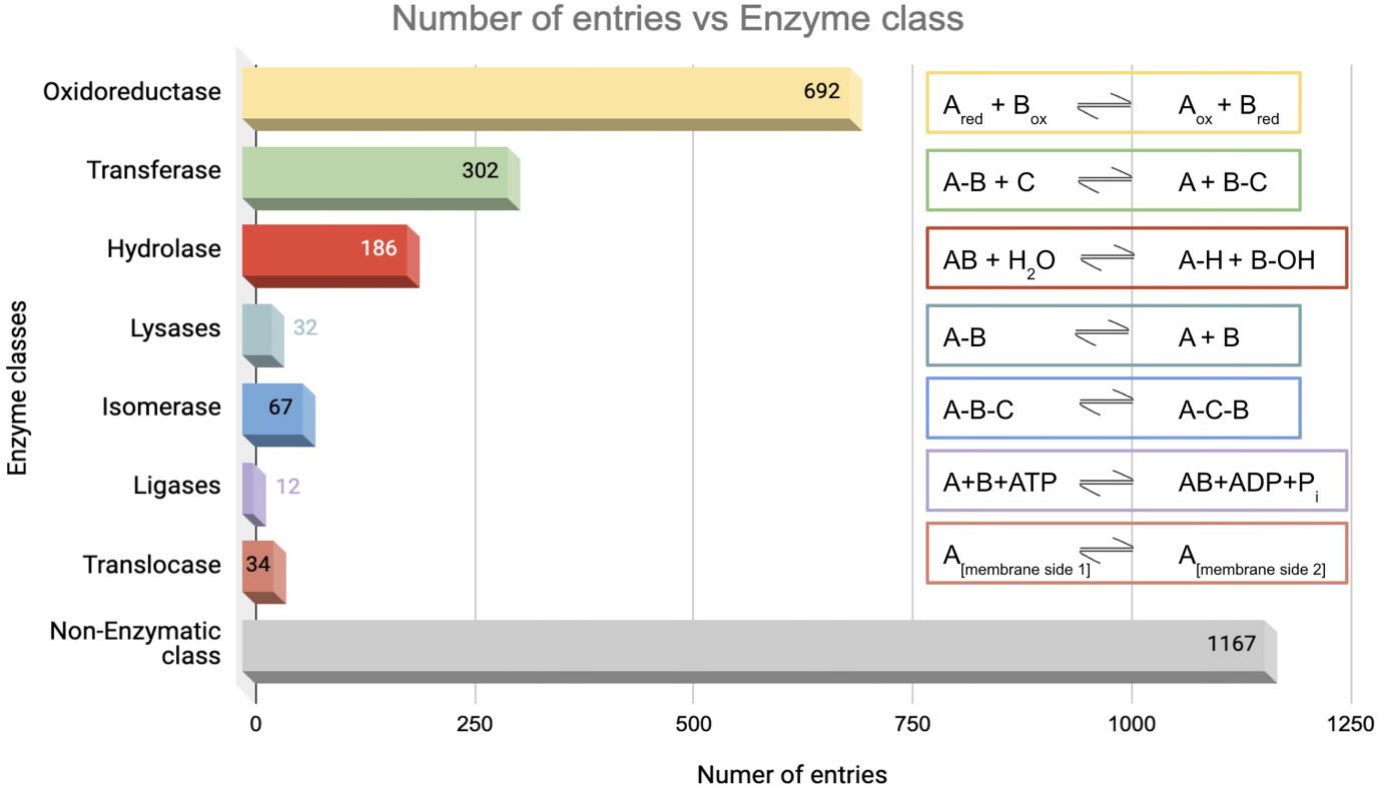
Distribution of enzyme classes in this database. Numbers of entries in each category are shown. The reactions catalysed by the respective enzyme classes are shown on the right-hand side.

The database was further classified based on enzyme classes (Fig. 7). The majority of the protein entries fall in the non-enzymatic class (*n* = 1167). Enzyme class 1, oxidoreductase (*n* = 692), exhibited the second highest number of entries, presumably because enzymes involved in redox activities are also associated with ROS [91]. Enzyme class 2, transferases, has the next largest number of entries (*n* = 302), which catalyse the transfer of functional groups across the molecules. Glutathione S-transferase is an example that catalyses peroxide to water or alcohol and oxidizes glutathione (GSH) to GSSG [92]. Enzyme class 3, hydrolases (*n* = 186), catalyse hydrolysis reactions. Epoxide hydrolase 1 (Ephx1) or microsomal (mEH) is an example that catalyses detoxification of genotoxic substances and scavenges ROS [93]. Enzyme class 4, lyases (*n* = 32), catalyses the lysis of bonds; an example of ROS involvement of lyase—ATP citrate lyase (ACLY) depletion elevates the intracellular ROS concentration [94]. Enzyme class 5, isomerases (*n* = 67), catalyses isomerization reactions. An example of ROS involvement of isomerase—protein disulfide isomerase, a thioredoxin family protein, is involved in redox-dependent activities, such as TNFα-dependent angiogenesis and indirect ROS production [95]. Enzyme class 6, ligases (*n* = 12), catalyses the joining of two molecules to form a single molecule. An example of ROS involvement of ligase—the E6AP E3 ubiquitin ligase, involved in the control of cell development and stress response, thus modulating cellular ROS levels [96]. To note, the E3 ubiquitin-protein ligase is omnipresent across Animalia and Plante kingdoms. For example, E3 ubiquitin-protein ligase, parkin, (from Kingdom Animalia, *Mus musculus* (mouse)) regulates cyclin E during neuronal apoptosis, which enhances cell viability and protects against oxidative stress [97]. Whereas E3 ubiquitin-protein ligase, (from Kingdom Plante, *Arabidopsis thaliana*) increases ABA-induced stomatal closure, ROS generation, and drought tolerance [98]. Enzyme class 7, translocases (*n* = 34), catalyses the movement of molecules or ions across cell membranes. An example of ROS involvement of translocase—peroxisome proliferator-activated receptor-γ coactivator 1-α (PGC-1α) enhances antioxidant gene expression thus reducing ROS formation in endothelial cells, and subsequent reduction in cell death [99].

### ROS-related diseases in the database

**Figure 8.**
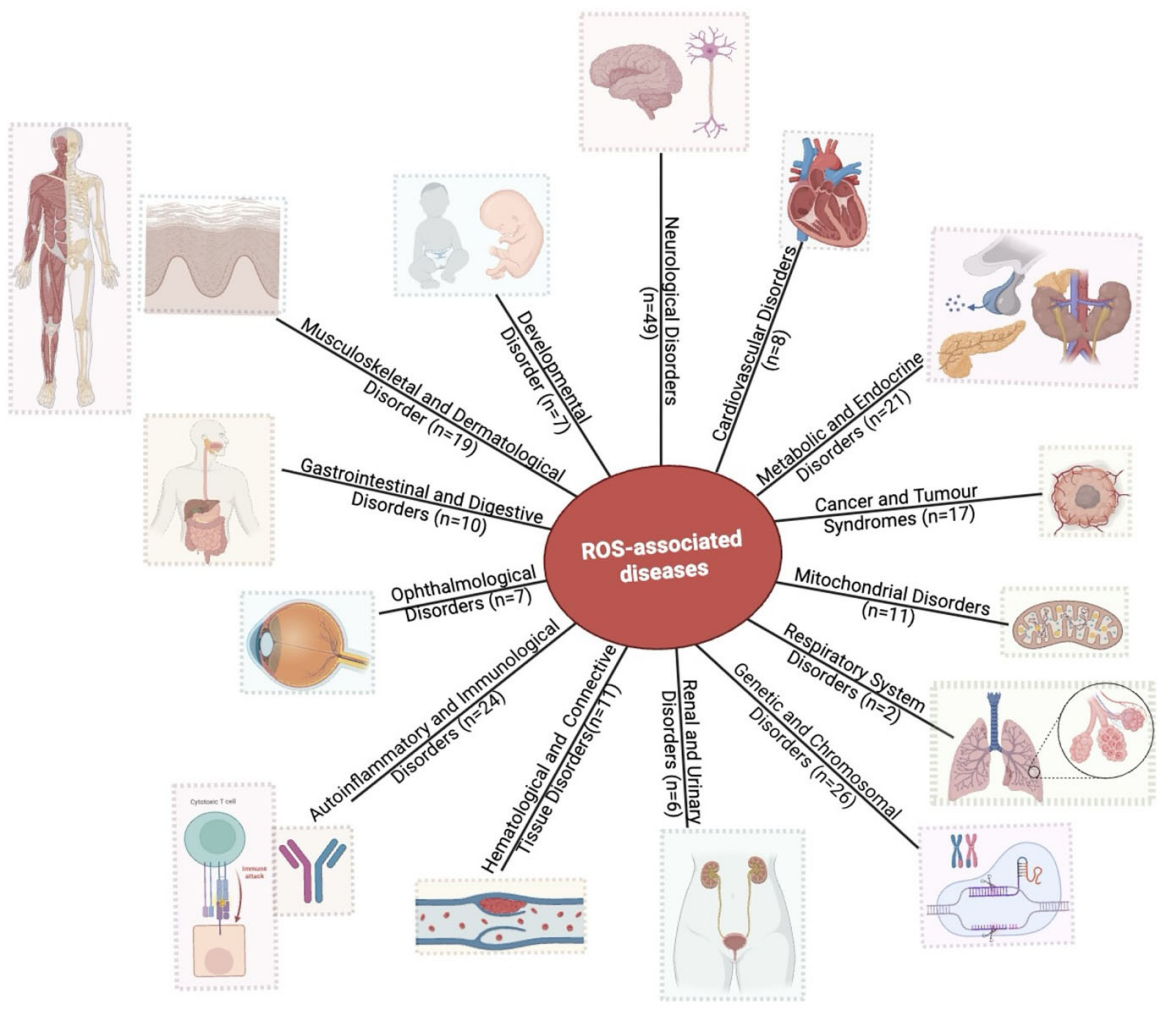
Distribution of disease categories (based on disease anatomy) in this database. Numbers of entries in each category are shown.

The database has 143 protein entries related to diseases. Individual protein entries were often associated with more than one disease, resulting in 218 disease entries. Based on the disease anatomy, these entries were categorized into 14 groups (Fig. 8). The disease categories included neurological disorders (*n* = 49), genetic and chromosomal disorders (*n* = 26), metabolic and endocrine disorders (*n* = 21), autoinflammatory and immunological disorders (n = 24), musculoskeletal and dermatological disorders (*n* = 19), cancer and tumour syndromes (*n* = 17), mitochondrial disorders (*n* = 11), haematological and connective tissue disorders (*n* = 11), ophthalmological disorders (*n* = 7), developmental disorders (*n* = 7), renal and urinary disorders (*n* = 6), cardiovascular disorders (*n* = 8), gastrointestinal and digestive disorders (n = 10), and respiratory system disorders (*n* = 2).

These diseases were further characterized based on ROS related four protein categories. Three disease categories, genetic and developmental diseases, brain and nervous system, and immunological disorders, were the most frequently involved in ROS production and scavenging. A few examples are, (i) paragangliomas 1, an autosomal dominant disorder, under oxidative stress, produces ROS and contributes to the formation and progression of tumours [100], (ii) ciliary dyskinesia (CILD48), an immunological or auto-immune disease, manifests impaired respiratory cilia motility leading to chronic respiratory problems and recurrent infections, affected by ROS [101], and (iii) ectodermal dysplasia with facial dysmorphism and acral, ocular, and brain anomalies, a brain and nervous system disorder, under oxidative stress, damages neurons and contributes to the neurodevelopmental abnormalities [102].

**Figure 9.**
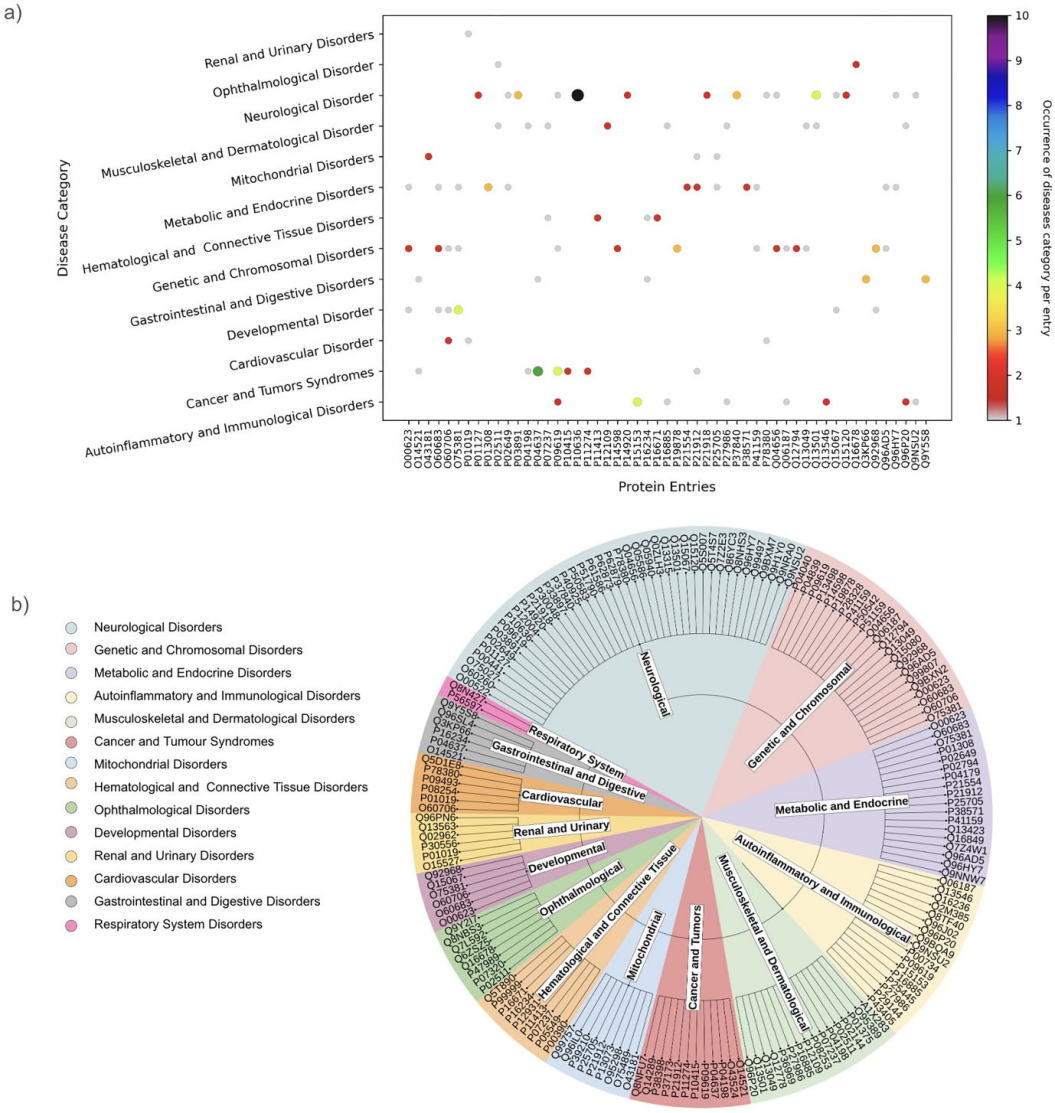
(a) Disease categories per protein entry (UniProt ID). (b) Pie chart representation of each disease category and corresponding UniProt IDs.

As mentioned above, one protein may be linked to multiple diseases. This has been demonstrated through the involvement of one UniProt ID in multiple diseases or disease categories (Fig. 9a). UniProt IDs corresponding to different disease categories are shown (Fig. 9b). Here, we have illustrated it using two examples, (i) Tauopathy: microtubule-associated protein tau is unable to bind microtubules in Tauopathy (a group of neurodegenerative disorders characterized by the pathological accumulation of abnormally phosphorylated tau protein in the brain) [103]. In this study, we demonstrate involvement of tau protein (UniProt ID: ‘P10636’) in multiple neurological disorders such as alzheimer’s, Parkinson’s, frontotemporal dementia, pick disease, corticobasal degeneration, and progressive supranuclear palsy 1. Tau protein (UniProt ID: P10636) in this database corresponds to eight diseases belonging to the ‘neurological disorder’ category (Fig. 9a).

(ii) The second example is disorder in platelet-derived growth factor receptor beta (PDGFRβ) (UniProt ID: ‘P09619’). This cell surface protein is involved in various cellular activities upon binding to PDGF ligands [104], including blood vessel formation by guiding the recruitment of pericytes and smooth muscle cells [105], kidney and cardiovascular development [106], etc. Mutations or chromosomal rearrangements activate abnormal PDGFRβ levels. It promotes proliferation of connective tissues, leading to chronic myelomonocytic leukaemia, juvenile myelomonocytic leukaemia, and acute myeloid leukaemia [107], myeloproliferative eosinophilic syndrome through clonal eosinophil expansion [108], idiopathic basal ganglia calcification 4 [109], etc. Germline mutations in PDGFRβ can produce benign tumours in skin, muscle, and bone in infantile myofibromatosis 1 [110], and Kosaki overgrowth syndrome, a developmental disorder, marked by tissue overgrowth and intellectual disability [111]. In this study, UniProt ID: ‘P09619’ was identified under two disease categories, namely, ‘Cancer and Tumors Syndromes’ and ‘Autoinflammatory and Immunological disorders’ (Fig. 9a).

The categorization of disease-induced ROS activities was not reported earlier; to the best of our knowledge, this is documented in this database for the first time. Documentation of these ROS-related diseases could presumably aid researchers in improving ROS-related therapeutics.

## Discussion

### Role of Cys-PTMs on ROS activities

As discussed above, site-specific oxidation or reduction of protein-cysteine plays an important role in ‘Oxidative eustress’, mediated via adaptive cell responses to ROS. Literature reports had also indicated the role of S-Glutathionylation and S-nitrosylation in inhibiting dehydrogenases, such as, alpha-KGDH, PDH, etc. Protein redox switches, i.e. Cys-PTMs, were mentioned as one of the four pivots of the ‘Redox Code’. Here, in this database, Cys-PTMs related to ROS are consolidated for a total of 1985 cysteine entries; the maximum of those are metal-binding (Table 2). Twelve different types of cysteine metal-binding are recorded (Table 3). The highest number of metal-binding cysteines was observed in Fe(II), predominantly present in peroxidase (96), and catalase (23). Fe(II) scavenges ROS (mainly H_2_O_2_) via Fenton reaction (Fe²⁺ + H₂O₂ → Fe³⁺ + •OH + OH^–^) [112]. Zn(II) ion (*n* = 192) are part of Zn-superoxide dismutase (Zn-SOD; *n* = 39), an antioxidant enzyme, that reduces superoxide (O_2_^−^) to peroxides (H_2_O_2_) [113]. Out of 13 K(I) ions, 12 belong to ascorbate peroxidase, which reduces peroxides to water [114, 115]. Out of 18 Mn(II) ions, six are part of Mn-superoxide dismutase, thus involved in scavenging ROS [116]. Mo(II) (*n* = 98) ions are involved in superoxide production during purine metabolism [117]. A total of 81 Mo(II) ions are part of ‘Protein-methionine-sulfoxide reductase’ (Msr), a thiol-dependent oxidoreductase, that reduces oxidized methionine using thioredoxin [118]. Iron–sulphur clusters are the co-factors that mediate electron transfer across the mitochondrial respiratory complexes and participate in ROS production by the Complexes I, II, and III [119]. In this database, 25 2Fe–2S and 5 4Fe–4S are reported.

**Table 2.** Statistics of cysteine post-translation modifications in this database.

Cysteine-post translational modifications (PTM)	Number of entries	Reactions
Metal-binding (−S-M^n+^)	1113	Prot-SH + M^+^ → Prot-S—M
Disulfide (−S = S−)	761	Prot-SH + P'SH → Prot-S = S-Prot' + H_2_O
Palmitoylation (−S-(CH_2_)_n_COOH)	55	Prot-SH + -(CH_2_)_n_COOH → Prot-S-(CH_2_)_n_COOH
Sulfenylation (−SOH)	27	Prot-SH + H_2_O_2_ → Prot-SOH + H_2_O
Nitrosylation (−SNO)	18	Prot-SH + NO• → Prot-SNO + H^+^
Thioester (−S-COR)	8	Prot-SH + -COR → Prot-S-COR
Glutathionylation (−SSG)	3	Prot-SH + GSSG → Prot-SSG + GSH

**Table 3.** Twelve different types of Cys-PTM, metal binding modifications present in this database.

Cysteine PTM metal modifications	Number of entries	Proteins involved (number of entries in this database)
Fe(II)	217	Peroxidase (96), Cytochrome (ETC transmembrane protein) (49), Myoglobin (39), Catalase (23)
Zn(II)	192	Super oxide dismuatase (SOD) (39)
Ca(II)	113	Protein kinase (14), Hedgehog (13)
Mo(II)	98	Protein-methionine-sulfoxide reductase (Msr) (81), Aldehyde oxidase (10), Xanthine oxidase (7)
Mg(II)	57	ATP synthetase (21)
2Fe–2S	25	CDGSH iron–sulphur domain-containing protein (3), NADH-quinone oxidoreductase (2), Succinate dehydrogenase iron–sulphur subunit (2), Xanthine oxidase (7), Aldehyde oxidase (9)
Mn(II)	18	Super oxide dismutase (SOD) (6), Arginase (3)
K(I)	13	Ascorbate peroxidase (12)
4Fe–4S	5	NADH-quinone oxidoreductase (2), Succinate dehydrogenase iron–sulphur subunit (2)
Na(I)	3	P2X purinoceptor 7 (P2 × 7) (ATP receptor) (P2Z receptor) (Purinergic receptor) (3)
Ni(II)	3	Urease (3)
Cu(I)	1	Copper transfer ATPase (1)—ATP-driven copper [Cu(+)] ion pump

### ROS interactions with cellular processes

**Figure 10.**
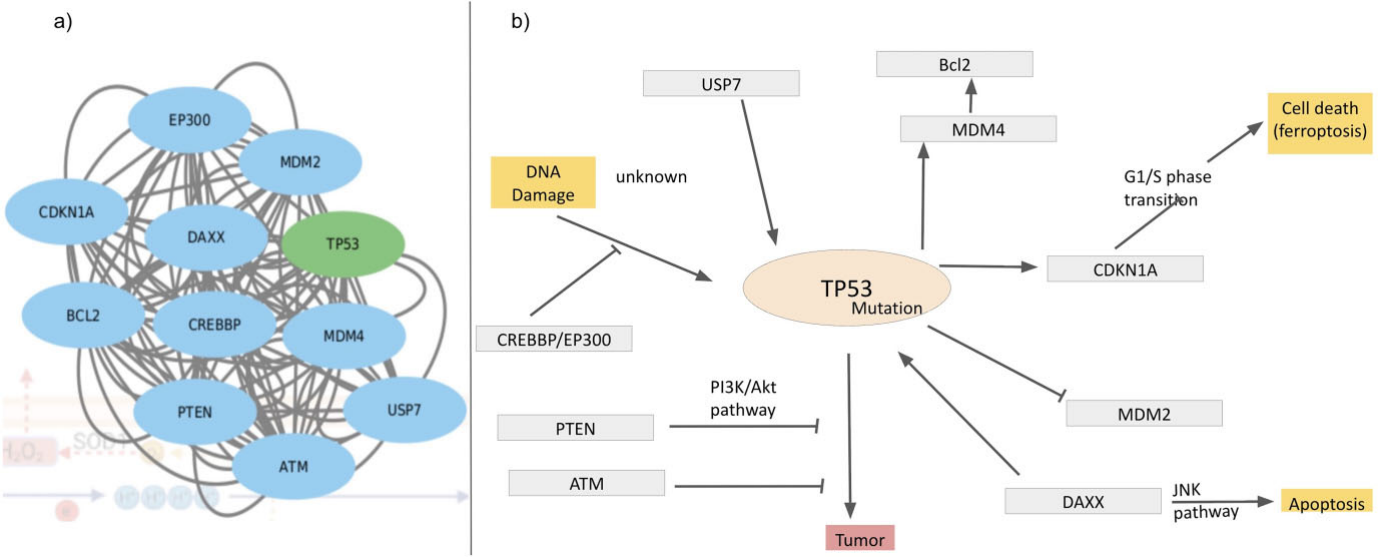
Interactome around Tp53, (a) derived from the STRING network and (b) regulatory pathways of Tp53; inhibition (—|) and interaction (→).

As mentioned in the introduction, ROS has double effects on cellular processes, depending on the ROS concentrations, a) physiological concentration of ROS leads to ‘oxidative eustress’ and overproduction leads to cell damage, i.e. ‘oxidative distress’. Here, we discuss a specific example describing ROS interactions with cellular processes, mainly apoptosis and DNA damage via overexpression of the cellular tumour antigen p53 (tumour suppressor p53), UniProt ID: P67939. The possible mechanisms of p53-induced apoptosis include DNA damage [120], deregulation of oncogene expression [121], ribonucleotide depletion [122], hypoxia [123], oxidative stress [124], and ferroptosis [125, 126]. Over half of tumour patients have TP53 mutations. Ferroptosis, an emerging form of cell death, is associated with the accumulation of Fe(II) and ROS. Query search with this UniProt ID resulted in the interactome around the Tp53 protein (Fig. 10a). Mutations of this tumour oncogene trigger various cellular pathways; the factors, upregulated or downregulated in this process, are captured in this interactome and are shown (Fig. 10b). Mutated p53 suppresses anti-oxidant activity in the cell, exerting higher levels of ROS, i.e. oxidative distress [127]. Under physiological conditions, Tp53 level is very low, as it is continuously degraded by **MDM2** protein [128]. Mutated Tp53 increases its inability to bind MDM2, thus expressing a tumour. **MDM4** is a regulator of the p53 growth-arresting function, in contrast to MDM2, the p53 apoptotic function regulator. After **DNA damage**, MDM4 is downregulated by dissociation of deubiquitylating enzyme, **USP**, from the MDM4–MDM2. On a lethal type of stress, a fraction of MDM4 residing in mitochondria recruits phosphorylation of p53 on Ser46; the phosphorylated p53 interacts with **BCL2** and ultimately releases cytochrome C; thus, MDM4 acts as a positive regulator of p53‐intrinsic apoptosis. Several other factors also interact with Tp53 and contribute towards tumour suppression. **PTEN** distantly regulates tumour suppression by inhibiting PI3K/Akt pathway [129]. Activated p53 upregulates cyclin dependent kinase inhibitor 1 A transcription [130, 131]. Thus, the database provides comprehensive interaction maps of different ROS-related proteins.

### Retrieval of the information from the database

**Figure 11.**
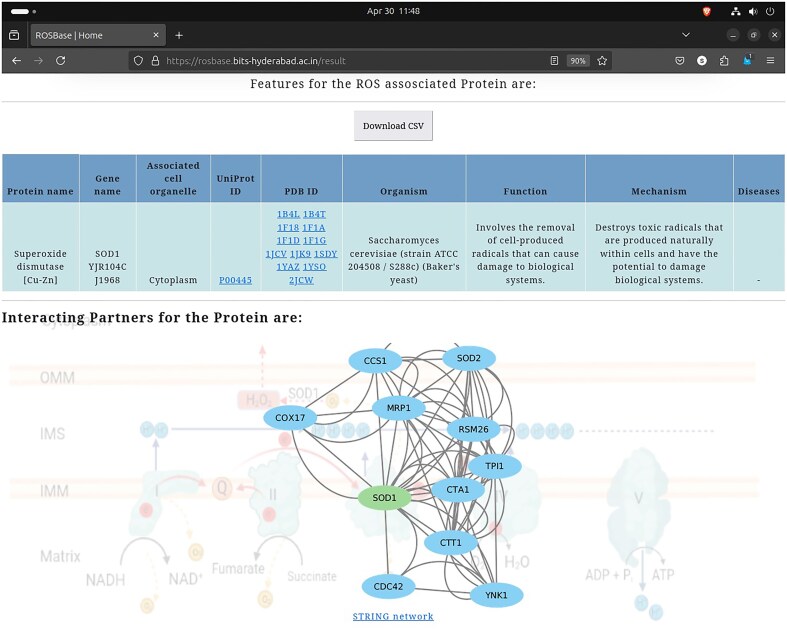
Database example output against the query—UniProt ID: ‘P00445’; returned in a tabular format (upper panel). Cytoscape-generated interactome image (lower panel).

User-specific information is retrievable from the webserver ROSBase (https://rosbase.bits-hyderabad.ac.in/) using different keywords (such as a protein name, UniProt ID, PDB ID, cell organelle, or gene name). For example, if a user provides the query as UniProt ID ‘P00445,’ the programme will search the JSON file (database) and return the output, in a tabular form (Fig. 11). In case of a single entry, the output would return an interactome image, (generated by Cytoscape software) obtained from the STRING database [132]. The interactome image has potential use in deciphering genetic interactions, protein–protein interactions, signalling pathways etc.

### Browser and operating system testing

The web server was rigorously tested across several browsers, namely, Chrome, Firefox, and Safari, to check compatibility and performance consistency. The testing was done on different operating systems (OSs), namely, Windows, Linux, and macOS, ensuring that performances were independent of the platform. The web server was also optimized for mobile viewing, with responsive design, thus it worked independent of the device settings.

### Long term database security and maintenance

To maintain, update, and sustain the database over time, scheduled data backups, performance monitoring via query profiling, and security updates are performed. For long-term database maintenance, scheduled backups and data retention for point-in-time recovery are performed. Regular upgradation and security patches are maintained to ensure security, compatibility, and the addition of new features. Dependencies, such as database drivers and libraries, are regularly updated to maintain compatibility and stability of the database. System sustainability and efficiency are maintained via the tools, such as, ‘Nginx’, the reverse proxy, ‘Gunicorn’, the process manager, and ‘Supervisor’, the automatic monitor for log generation, reporting errors, and uptime, server restart, etc.

## Conclusion

ROS is an obligatory byproduct of various cellular processes across the taxonomic world which participate in both physiology and pathology. ROS-related information, such as ROS production, scavenging, regulation of several biochemical processes, diversity across the taxonomy, disease involvements etc., was scattered in the literature; however, those were not consolidated for ready reference. ROSBase1.0 is the first database of its kind that stores and disseminates ROS-related information. This database returns the following information in retrievable formats: (i) protein name, (ii) cell organelle, (iii) UniProt ID, (iv) PDB ID, (v) gene name, (vi) function, (vii) ROS-associated mechanism, (viii) diseases, and (ix) interactome image. The search keywords are any of the following: protein name, cell organelle, UniProt ID, PDB ID, and gene name. This database, for the first time, annotated the taxonomic world, diseases etc., in terms of their ROS-related activities. It has quantitatively highlighted that lower organisms contributed more towards ROS scavenging compared to ROS production. Within higher organisms, plants were more involved in ROS scavenging and ROS-directed physiology compared to ROS production. The role of ROS in various diseases was also quantified in this database. Three disease categories (anatomy-based) were maximally involved with ROS activities. This information gathered in the database has the potential utility towards better therapeutics for ROS-related diseases.
